# Restoration of Sarco/Endoplasmic Reticulum Ca^2+^-ATPase Activity Functions as a Pivotal Therapeutic Target of Anti-Glutamate-Induced Excitotoxicity to Attenuate Endoplasmic Reticulum Ca^2+^ Depletion

**DOI:** 10.3389/fphar.2022.877175

**Published:** 2022-04-20

**Authors:** Wen Zhang, Fanghua Ye, Nan Pang, Miriam Kessi, Juan Xiong, Shimeng Chen, Jing Peng, Li Yang, Fei Yin

**Affiliations:** ^1^ Department of Pediatrics, Xiangya Hospital, Central South University, Changsha, China; ^2^ Hunan Intellectual and Developmental Disabilities Research Center, Pediatrics, Changsha, China; ^3^ Clinical Research Center for Children Neurodevelopmental Disabilities of Hunan Province, Xiangya Hospital, Central South University, Changsha, China; ^4^ Kilimanjaro Christian Medical University College, Moshi, Tanzania

**Keywords:** SERCA2b, excitotoxicity, calcium depletion, endoplasmic reticulum stress, mitochondria, CDN1163

## Abstract

Glutamate-induced excitotoxicity is a pathological basis of many acute/chronic neurodegenerative diseases. Sarco/endoplasmic reticulum Ca^2+^-ATPase (SERCA2b) is a membrane-embedded P-type ATPase pump that manages the translocation of calcium ions (Ca^2+^) from cytosol into the lumen of the endoplasmic reticulum (ER) calcium stores. It participates in a wide range of biological functions in the central nervous system (CNS). However, the role of SERCA2b in glutamate-induced excitotoxicity and its mechanism must be elucidated. Herein, we demonstrate that SERCA2b mutants exacerbate the excitotoxicity of hypo-glutamate stimulation on HT22 cells. In this study, SERCA2b mutants accelerated Ca^2+^ depletion through loss-of-function (reduced pumping capacity) or gain-of-function (acquired leakage), resulting in ER stress. In addition, the occurrence of ER Ca^2+^ depletion increased mitochondria-associated membrane formation, which led to mitochondrial Ca^2+^ overload and dysfunction. Moreover, the enhancement of SERCA2b pumping capacity or inhibition of Ca^2+^ leakage attenuated Ca^2+^ depletion and impeded excitotoxicity in response to hypo-glutamate stimulation. In conclusion, SERCA2b mutants exacerbate ER Ca^2+^-depletion-mediated excitotoxicity in glutamate-sensitive HT22 cells. The mechanism of disruption is mainly related to the heterogeneity of SERCA2b mutation sites. Stabilization of SRECA2b function is a critical therapeutic approach against glutamate-induced excitotoxicity. These data will expand understanding of organelle regulatory networks and facilitate the discovery and creation of drugs against excitatory/inhibitory imbalance in the CNS.

## Introduction

Glutamate is the primary excitatory neurotransmitter in the mammalian central nervous system (CNS). Glutamatergic neurotransmission controls many cognitive, motor, sensory, and autonomic activities. In addition, glutamate is critical in maintaining the balance between excitation and inhibition in the CNS (Meldrum, 2000). Glutamate is released from glutamatergic neurons into the synaptic gap and transmits excitatory output by binding to ionotropic or metabotropic receptors. High-affinity transport proteins expressed in neurons and glial cells are responsible for rapidly removing the glutamate from the synaptic gap to the astrocytes. Astrocytes then convert glutamate to inert glutamine by intracellular glutamine synthetase (GS) and release it into the synaptic gap for re-uptake by neurons in what is known as the “glutamate–glutamine” cycle (Hayashi, 2018). Glutamate accumulation at the synapse exceeding the physiological range is detrimental. Too much glutamate released at the synapse results in intracellular calcium-ion (Ca^2+^) overload, which leads to neuronal excitotoxicity and subsequent neuronal dysfunction and apoptosis (Angelova et al., 2019).

Impaired glutamate homeostasis has severe neuropathological consequences and is associated with a variety of CNS disorders, such as epilepsy, Alzheimer’s disease (AD), Huntington’s disease, and Parkinson’s disease (Hynd et al., 2004; Andre et al., 2010; Eid et al., 2016; Zhang et al., 2019; Rezaeian et al., 2022). Neuroprotective strategies for glutamate receptors and transporters include targeting ion-channel pores, glutamate binding sites, and glycine binding sites. For example, non-competitive N-methyl-D-aspartic acid receptor (NMDAR) antagonists, such as aptiganel hydrochloride, dizocilpine, memantine, and dextromethorphan, have shown neuroprotective effects in cultured neurons and animal models (Kroppenstedt et al., 1998; Pu et al., 2015; de Miranda et al., 2017; Liu et al., 2020). However, only memantine has shown neuroprotective effects in clinical trials (Brown et al., 2013). The competitive NMDAR antagonist (selfotel) increases the mortality rate in acute ischemic stroke patients (Davis et al., 2000), and SDZ EAA 494 has been reported to cause memory impairment and to have no clinical benefit in traumatic brain injury patients (Slieker et al., 2008). Targeting excitatory amino acid transporter 2 (EAAT2), the major glutamate transporter in the CNS, is a novel approach for developing epilepsy therapy (Ngomba and van Luijtelaar, 2018; Green et al., 2021). However, the functional characteristics of the glutamate transporter and its implications for epileptic neuropathology and behavior require further study (Zaitsev et al., 2020). Excitotoxicity can lead to neuronal death through multiple death pathways and targets. Therefore, it is essential to explore the potential excitotoxic mechanisms for developing neuroprotective drugs.

Ca^2+^ is an essential cellular signaling regulator that acts as a crucial second messenger in many cellular processes and “enforcers” of glutamate excitotoxicity. The activation of the glutamate receptors leads to an inward flow of extracellular Ca^2+^ and an increase in intracellular Ca^2+^ levels ([Ca^2+^]_cyto_). The elevated amount of [Ca^2+^]_cyto_ further triggers the opening of Ca^2+^-releasing channels in the endoplasmic reticulum (ER), activating inositol 1,4,5-trisphosphate receptors (IP3Rs) and ryanodine receptors (RYRs). This process is known as calcium-induced calcium release (CICR), which is a primary mechanism of calcium signal generation and amplification in the cells (Verkhratsky and Shmigol, 1996). Inefficient removal of [Ca^2+^]_cyto_ may affect numerous Ca^2+^-dependent biological functions, ultimately leading to cellular dysfunction and neuronal death. There are several hypotheses for this phenomenon. The downstream effects of increased levels of the [Ca^2+^]_cyto_ are mitochondrial Ca^2+^ overload via mitochondrial Ca^2+^ uniporter protein (MCU). Mitochondrial Ca^2+^ overload results in increased mitochondrial membrane permeability, imbalance of redox reaction, release of reactive oxygen species (ROS), and release and activation of apoptosis-related factors, all of which are associated with glutamate-induced neuronal death (Nicholls and Budd, 1998; Plotegher et al., 2021). Another possible explanation is that impaired levels of [Ca^2+^]_cyto_ clearance lead to ER Ca^2+^ depletion. Since chaperones require high concentrations of Ca^2+^ and ATP in the process of protein folding, excessive unfolded proteins or misfolded proteins accumulate in the ER lumen, causing endoplasmic reticulum stress (ERS) (Hetz and Saxena, 2017; Ghemrawi and Khair, 2020). ERS can cause cells to produce adaptive responses, including reducing protein translation, enhancing degradation of unfolded proteins, and folding chaperone proteins, a process known as unfolded protein response (UPR) (Ghemrawi and Khair, 2020). ER Ca^2+^ depletion pushes UPR out of balance, leading to forms of cell death such as apoptosis and autophagy. In glutamate-induced neuronal toxicity, the interactions between [Ca^2+^]_cyto_, ER Ca^2+^, mitochondrial Ca^2+^, UPR, and mitochondrial dysfunction require further investigation.

Sarco/endoplasmic reticulum Ca^2+^-ATPases (SERCA2b) pumps are membrane-embedded P-type ATPases pumps that mediate the translocation of [Ca^2+^]_cyto_ to the ER lumen by hydrolyzing ATP against a concentration gradient (Chen J. et al., 2020). *ATP2A2* encodes SERCA2a–c and is widely distributed in various cells. SERCA2b is the most widely expressed pump in brain tissue (Baba-Aissa et al., 1998; Mata and Sepúl`veda, 2005), including in the soma, dendrites, and axon terminals (Hartter et al., 1987; Lysakowski et al., 1999; Bouchard et al., 2003). There is growing evidence that *ATP2A2* mutations are associated with neuropsychological disorders such as epilepsy, mild intellectual disability, bipolar disorder, and schizophrenia (Cederlöf et al., 2015; Britzolaki et al., 2018; Gordon-Smith et al., 2018), but the *in vivo* significance of SERCA2b in neurons and the brain has not been extensively studied (Nakajima et al., 2021). It is understood that excessive glutamate leads to cell toxicity, but it remains unknown whether low amounts of glutamate can also impair cell functions via ER Ca^2+^ depletion, an increase in levels of [Ca^2+^]_cyto_, and induction of mitochondrial toxicity. Since SERCA2b has a vital role in regulating neuronal Ca^2+^ homeostasis, we speculate that SERCA2b is involved in glutamate-induced excitotoxicity processes by regulating ER Ca^2+^ storage. We also hypothesize that the loss-of-function (LOF) mechanism of *ATP2A2* mutations demonstrated in Darier’s disease (Ahn et al., 2003) can explain the relationship between ER Ca^2+^ storage and glutamate-induced excitotoxicity.

We built glutamate excitotoxicity models of HT22 cells transfected with four SERCA2b mutants (G23R, D567Y, G860S, and I1014V). The G806S mutant has been published previously (Peng et al., 2018), whereas the other three mutants were from our in-house database of genetic epilepsy, a typical excitotoxic pathogenic disease. We found that the SERCA2b mutants exacerbated ER Ca^2+^ depletion, led to mitochondrial Ca^2+^ overload, and increased ERS and mitochondria-mediated apoptosis induced by low glutamate concentration. The mechanism of ER Ca^2+^ depletion depended on the effect of different mutation sites on calcium channels, including pumping capacity and passive leakage. Rescue of ER Ca^2+^ depletion facilitated the suppression of excitotoxicity. These results expand the knowledge of glutamate-induced excitotoxicity.

## Materials and Methods

### Cell Culture and Transfection

HT22 cells were obtained from Kunming Cell Bank of the Chinese Academy of Sciences (Kunming, China), and they were cultured in DMEM medium (Hyclone, USA) supplemented with 10% FBS (Gibco, USA), 100 U/ml penicillin, and 100 μg/ml streptomycin (Gibco, USA). Cell culture was performed at 37°C in an incubator filled with 5% CO_2_ and 95% air. Glutamate (0.5, 1, 2, 5, 10, 20 mM) (Sigma, USA) was applied to HT22 cells 24 h for subsequent analysis. According to the DNA transfection protocol, the SERCA plasmids were transfected into HT22 cells by jetOPTIMUS (Polyplus transfection, France). The control group (CON) mentioned in this article refers to the cells transfected with the empty plasmid (pcDNA3.1) only. The cells were analyzed for the glutamate response 24 after transfection.

### Mutant SERCA2 Plasmid Construct

The pcDNA3.1-SERCA2b-3xFLAG plasmid containing human SERCA2b was purchased from GenScript. Site-directed mutagenesis was performed using the QuickMutation™. After sequencing confirmation of the wildtype SERCA2b, all mutants (G23R, D567Y, G860S, I1014V) studied were induced by site-directed mutagenesis using Serca2b as a template and confirmed by sequencing again. Primers used for site-directed mutagenesis PCR are listed in Supplementary Table S1.

### Cell Viability Assay

Cell Counting Kit-8 assay (CCK-8): Cells were seeded into 96-well plates at a density of 5 × 10^3^ cells per well. After 12 h, cells in each well were incubated with 10 μl CCK-8 solution (Beyotime, China) for 2 h at 37°C. The incubation plates were then placed in a microplate reader (Biotek Synergy H1, USA) to determine the optical density (OD) values at 450 nm. The curves were plotted according to the OD values. Thiazolyl Blue Tetrazolium Bromide assay (MTT): Cells were seeded into 96-well plates at a density of 5 × 10^3^ cells per well. After 12 h, cells in each well were incubated with 10 μl MTT solution (Beyotime, China) for 4 h at 37°C. After that, 100 µl of Formazan solution (Beyotime, China) was added to each well, mixed properly, followed by incubation for another 4 h. Then the OD of each well was measured at 570 nm. Lactate dehydrogenase (LDH) release assay was performed using the LDH Cytotoxicity Assay Kit (Beyoutime, China). Briefly, cells were plated in a 96-well plate, and 60 μl of LDH assay working solution was added to each well according to the operation manual, mixed well, and incubated at room temperature (about 25°C) in a dark place for 30 min. Then the OD value of each well was measured at 490 nm. The absorbance after Triton X-100 treatment was regarded as A_max_. $\mathrm{Cell}\;\mathrm{activity}\left(\%\right)=\left[1-\left(A_{samples}-A_{con}\right)/\left(A_{\max}-A_{con}\right)\right]\times 100$ .

### Detection of Cytoplasmic and Mitochondrial Ca^2+^

Cells were incubated with 5 μM Fluo-4 AM (Invitrogen, USA) or Rhod-2 AM (Abcam, USA) for 20 min. After washing with PBS twice, the buffer was replaced with HBSS without Ca^2+^ and Mg^2+^. Cells were scanned and imaged at 5 s intervals under a microplate reader (Biotek Synergy H1, USA) by successive addition to a final concentration of 1 mM glutamate, 10 mM caffeine, and 5 mM EGTA. For the rescue of [Ca^2+^]_ER_ experiment, CDN1163 (10 μM, Selleck) or thapsigargin (2 μM, Sigma) was added and incubated for 10 min, followed by glutamate stimulation. Fluo-2 AM Ex/Em = 549/578 nm, Fluo-4 AM Ex/Em = 494/506 nm.

### Transmission Electron Microscope

Cells were fixed with 1% osmic acid for 2 h at 4°C and centrifuged at 200 rpm for 10 min; then, cell pellets were collected. The cell pellets were fixed again by 1% osmic acid and 0.1 M PBS (PH7.4) for 2 h at 20°C, followed by dehydration, infiltration, embedding, sectioning, and staining (2% Uranyl acetate and lead citrate). Finally, they were observed under an electron microscope (Hitachi, Japan).

### Mitochondrial Membrane Potential Detection

Cells were incubated with 10 nM tetramethylrhodamine, ethyl ester (TMRE) (Beyotime, China) for 30 min at 37°C at 24 h after glutamate stimulation. Then, cells were washed twice with a pre-warmed cell culture medium and observed in a confocal laser microscope (LEICA TCS SP8, Germany). Cells treated with 10 μM carbonyl cyanide-m-chlorophenyl hydrazone (Beyotime, China) were used as a positive control. Image J software was used for analyzing fluorescence intensity. Ex/Em = 550/575 nm.

### ROS Detection

Cells were incubated with 10 nM 2′,7′-Dichlorodihydrofluorescein diacetate (DCFH-DA) (Nanjing Jiancheng Bioengineering Institute, China) for 1 h at 37°C, and then centrifuged at 1,000 rpm for 5 min. The cell pellets were observed under a confocal laser microscope (LEICA TCS SP8, Germany). ImageJ software was used for analyzing fluorescence intensity. Ex/Em = 500/525 nm.

### Western Blotting

The cells were rinsed with ice-cold PBS two times before the total protein was extracted. The extraction process took place on ice. After adding RIPA buffer (50 mM Tris-HCl pH 7.5, 150 mM NaCl 1% sodium deoxycholate, 0.1% SDS, 2 mM EDTA) the homogenate was centrifuged at 12,000 rpm for 10 min at 4°C. For SERCA2 solubility analysis experiments, the addition of 1% Triton X-100 and repeated ultrasonic fragmentation helped lysis, and the supernatant was subjected to denaturation (98°C, 10 min) to enhance lysis. The bicinchoninic acid protein assay kit determined the protein concentration. Equal amounts of protein (20–40 μg) were separated by a 10%–15% SDS-PAGE gel and transferred onto a PVDF membrane. PVDF membrane was blocked with 5% skim milk at room temperature in TBST for 1 h. Membranes were incubated with primary antibodies (details in Supplementary Table S2) and horseradish peroxidase-conjugated secondary antibodies (Jackson ImmunoResearch). The membranes were then treated using an enhanced chemiluminescence kit (Millipore). Image J software (National Institutes of Health, USA) was used to analyze protein expression levels quantitatively.

### TdT-Mediated dUTP Nick-End Labeling Assay

TUNEL kit (C1088, Beyotime) was used to detect apoptotic cells according to the manufacturer’s instructions. In brief, samples were fixed with 4% PFA, permeabilized with 0.1% Triton X-100 in PBS for 10 min at room temperature, incubated with working solution (terminal deoxynucleotidyl transferase, TdT) for 1 h, and shielded from light at 37°C. The nuclei were then stained with the DAPI for 10 min. The specimens were analyzed using a confocal laser microscope (LEICA TCS SP8, Germany.

### Quantitative RT-PCR

Total RNA was isolated using TRIzol Lysis Reagent (Invitrogen), and cDNA was synthesized by All-in-OneTMFirst-Strand cDNA Synthesis Kit (GeneCopoeia, USA). For qPCR, amplification reactions were performed using All-in-One™qPCR Mix (GeneCopoeia, USA), run on the ABI-Ⅶ Real-Time PCR detection system. The primers were synthesized by Tsingke Company (China); the details of sequences are listed in Supplementary Table S3. All experimental steps were performed following the manufacturers’ instructions. Data analysis was performed using the 2^−(ΔΔCT)^ method to determine the relative quantitative level and was expressed as a fold-difference to the relevant control (recognized as 1 ± 0.00).

### Isolation of Microsomes

According to the modified Parsons’s protocol, microsomes (crude ER) were isolated by serial centrifugation (Parsons et al., 2000; Wang et al., 2011). Briefly, cells cultured in 6-well plates were transiently transfected and then collected and suspended with 350 ul of low-tonic buffer per well (10 mM Tris-HCl, pH 7.5, 0.5 mM MgCl, and 1 mM phenylmethanesulfonyl fluoride). The cells were homogenized for 45 strokes with a glass homogenizer and then made isotonic by adding 350 ul buffer (0.5 M sucrose, 0.3 M KCl, 6 mM β-mercaptoethanol, 40 uM CaCl2, 10 mM Tris-HCl, pH 7.5). The homogenate was centrifuged at 5,000 g for 10 min to yield a supernatant (S1) and a crude nuclear pellet. S1 was then centrifuged for 20 min at 18,000 g to yield a supernatant (S2) and a crude mitochondrial pellet (P2). S2 was then centrifuged for 3 h at 20,000 g to yield a cytosolic fraction (S3) and the crude microsomal pellet (P3). The P3 pellets (microsomes) were suspended in 65 μl buffer containing 10 mM Tris-HCl, pH 7.5, 0.15 M KCl, 0.25 M sucrose, 20 mM CaCl_2_and 3 mM β-mercaptoethanol. The concentration of microsomes was determined by the Lowry method.

### SERCA2 Activity

SERCA2 activity was measured by the inorganic phosphorus method (A070-4-2, Nanjing Jiancheng Bioengineering Institute). Briefly, ATP was decomposed into ADP and inorganic phosphorus by ATPase. The reagents were added separately, then mixed according to the protocol, and the reaction was carried out at 37°C for 10 min. After that, 100 ul of the sample was added and mixed for the enzymatic reaction. After centrifugation at 3,500 rpm for 10 min, 150 μl of the supernatant was collected to determine phosphorus. The absorbance was measured at A636 wavelength with 0.02 μmol/ml standard phosphorus solution as a reference. The amount of ATPase to produce 1umol of inorganic phosphorus per mg of microsomes per hour was defined as 1 ATPase activity unit (μmolPi/mg protein/hour).

### Statistical Analysis

The student’s t-test was used to determine significant differences between the two groups. One-way analysis of variance was utilized to determine significant differences among multiple groups. A *p*-value of less than 0.05 was considered statistically significant. Results were expressed as means ± SEM.

## Results

### SERCA2b Mutants Exacerbate Hypo-Glutamate-Induced ER Stress and Cell Death in HT22 Cells

**FIGURE 1:**
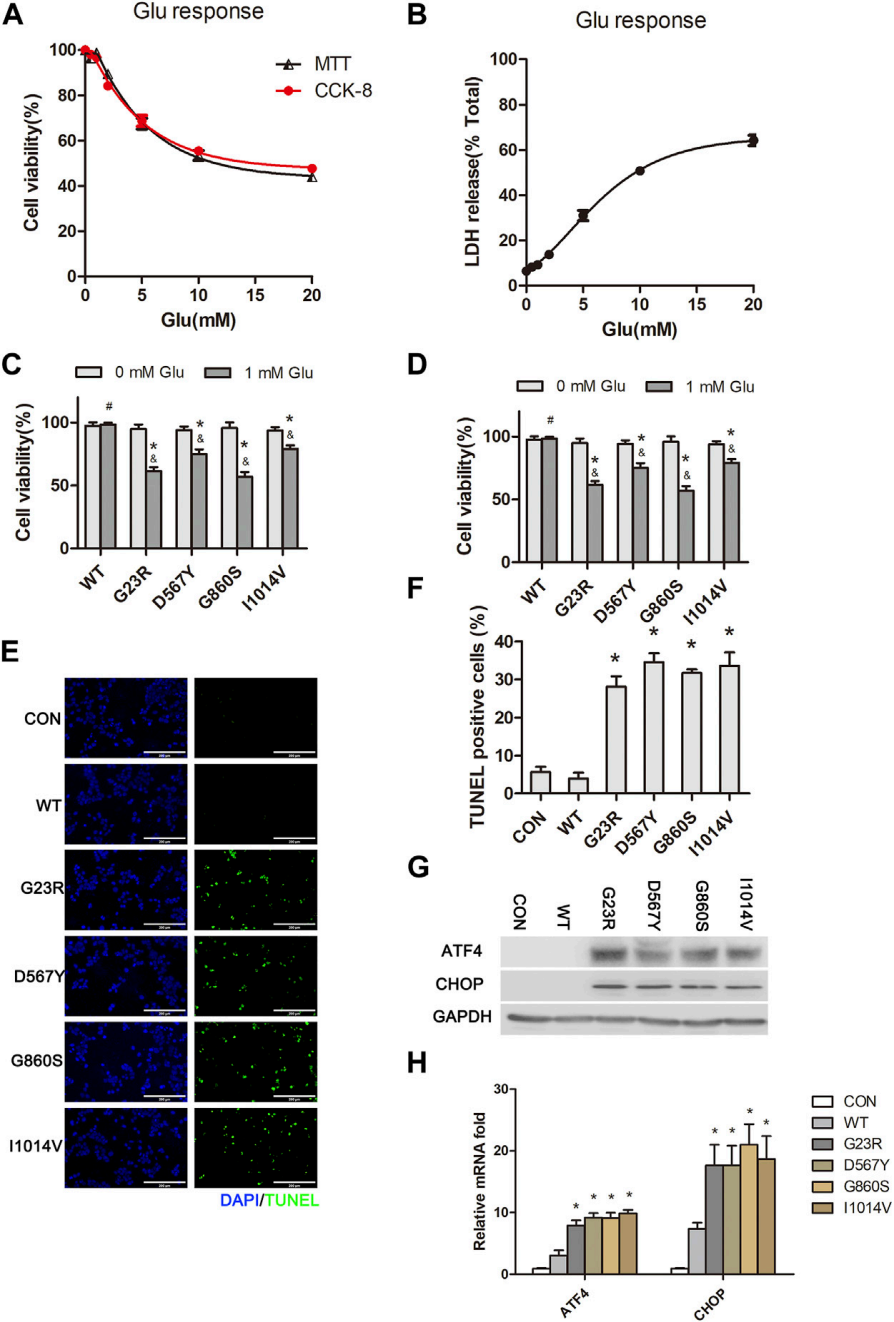
SERCA2 mutants exacerbate hypo-glutamate-induced ERS and cell death in HT22 cells. **(A,B)**: MTT assay, CCK-8 assay **(A)**, and LDH release assay **(B)** show decreased cellular activity in a glutamate concentration-dependent manner in native HT22 cells. **(C,D)**: CCK-8 assay **(C)** and LDH assay **(D)** show SERCA2 mutants exacerbated hypo-glutamate-induced cell death. *p < 0.05 compared with respective 0 mM glutamate stimulation. ^#^ *p* > 0.05 compared with respective 0 mM glutamate stimulation. ^&^ *p* < 0.05 compared with WT in 1 mM glutamate stimulation, respectively. **(E,F)**: Representative images **(E)** and summary data **(F)** show increased cell apoptosis of *SRCA2* mutants under hypo-glutamate stimulation by TUNEL staining. *p < 0.05 compared with WT group, respectively. Scale bar, 200 µm. **(G,H)**: Increased expression of ERS marker of indicated proteins **(G)** and mRNAs **(H)** under hypo-glutamate stimulation by Western blotting, real-time qPCR, respectively. *p < 0.05 compared with WT group.

Excitotoxicity plays an essential role in CNS injury. Intracellular calcium overload and mitochondrial dysfunction are the leading causes of high glutamate-induced neuronal death (Meldrum, 1993; Olloquequi et al., 2018). We tested the impacts of different concentrations of glutamate stimulation on HT22 cell viability using both CCk-8 and MTT assays and found that cell viability was reduced in a dose-dependent manner (Figure 1A). High concentrations of glutamate (5 mM) stimulation for 24 h resulted in reduced cell activity approaching 60%–70%. LDH release assays further confirmed the excitatory neurotoxic effect of excess glutamate (Figure 1B). Low glutamate stimulation concentration (1 mM) did not directly affect cell viability. However, in transfected SERCA2b mutants (G23R, D567Y, G860S, and I1014V), cell viability was significantly decreased after 24 h of stimulation with a low concentration of glutamate (Figure 1C). Furthermore, LDH release assays also confirmed the sensitivity of mutant cells to excitotoxicity of low glutamate (Figure 1D). TUNEL analysis revealed frequent apoptotic cells in all mutant groups, but few apoptotic cells were observed in the wildtype (WT) or CON group after 24 h of glutamate stimulation (Figures 1E,F). To explore the cause of apoptosis in HT222 cells, we evaluated the severity of ERS after 1-mM glutamate stimulation. It was found that low glutamate-induced apoptosis and protein kinase R-like ER kinase- C/EBP homologous protein (PERK-CHOP) signaling pathways were activated in mutant cells, which was confirmed by increased protein and mRNA levels of ERS markers (Figures 1G,H). In summary, these results suggest that the four SERCA2b mutants exacerbate the excitotoxicity induced by low concentrations of glutamate by affecting ERS.

### Reduced Pump Activity and Cytoplasmic Ca^2+^ Clearance Efficiency in SERCA2b Mutants

**FIGURE 2:**
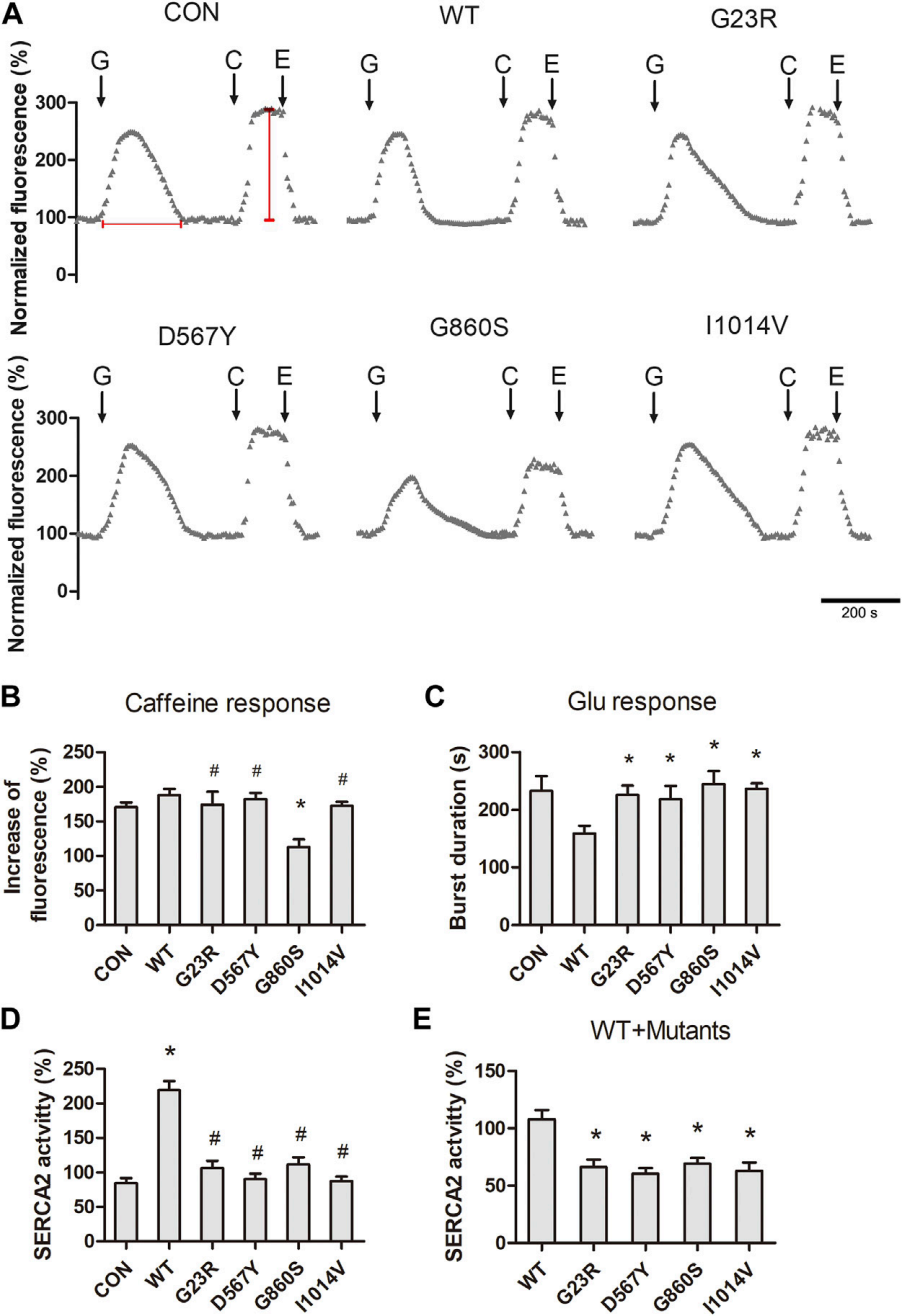
Declined pump activity and cytoplasmic Ca^2+^ clearance efficiency in SERCA2 mutants. **(A)** Representative [Ca^2+^]_cyto_ response of SERCA2 mutants. The addition of glutamate (G), caffeine (C), EGTA (E) is indicated with black arrows. Scale bar, 200 s. Horizontal and vertical red lines represent the level of the [Ca^2+^]_cyto_ clearance efficiency and [Ca^2+^]_ER_ storage, respectively. **(B)** Quantification of [Ca^2+^]_ER_ storage in response to caffeine. *p < 0.05 compared with WT group, ^#^ *p* > 0.05 compared with WT group, respectively. **(C)** Quantification of [Ca^2+^]_cyto_ clearance efficiency in response to glutamate. *p < 0.05 compared with WT group, respectively. **(D,E)**: Quantification of SERCA2 activity of mutants when transfected alone **(D)** or co-transfected **(E)**, SERCA2 mutants were inactive and even inhibited endogenous SERCA2 activity. **(D)**: *p < 0.05 compared with CON group, ^#^ *p* > 0.05 compared with CON groups respectively. **(E)**: *p < 0.05 compared with WT group.

Severe ERS is often caused by the depletion of ER Ca^2+^, as the process of proteins folding by the molecular chaperones requires high levels of Ca^2+^. We next measured the levels of [Ca^2+^]_cyto_ concentration in response to glutamate stimulation and ER Ca^2+^ storage levels. After 10 min of loading with Fluo-4 AM, cells stimulated with 1-mM glutamate showed a transient increase in calcium fluorescence signal and gradually returned to the resting levels. The magnitude of the glutamate response was significantly lower in the G860S mutant than in the WT group (Figures 2A–C). The [Ca^2+^]_cyto_ clearance efficiency was significantly lower in all groups than the WT group (Figures 2A–C), suggesting SERCA2b-mediated Ca^2+^ uptake dysfunction in mutants. Rapid addition of 10-mM caffeine was used to release ER Ca^2+^, and the results showed Ca^2+^ storage in G23R/D567Y/I1014V groups was similar to the WT group, which was significantly decreased in the G860S group (Figures 2A,B). We next assayed SERCA2b activity of isolated microsomes (including both the intrinsic activity of SERCA2b and exogenously transferred mutant activity). SERCA2b activity was significantly increased in the WT group compared with the CON group, while the activity was not significantly altered in the G23R/D567Y/G860S/I1014V groups (Figure 2D). However, when the WT and mutant plasmids were co-transfected (WT:mutants = 1:1), the SERCA2 activity of each mutant group decreased when compared with WT groups alone (Figure 2E). These results suggested that the SERCA2b mutants were inactive and might have affected the endogenous pump activity, which is consistent with the decreased [Ca^2+^]_cyto_ scavenging efficiency in the glutamate response. In summary, SERCA2b mutants lost the ability to pump Ca^2+^ against concentration, resulting in a slow [Ca^2+^]_cyto_ clearance responding to glutamate stimulation.

### Insoluble, Low-Expression Mutant Protein Causes Loss-of-Function of SERCA2b

**FIGURE 3:**
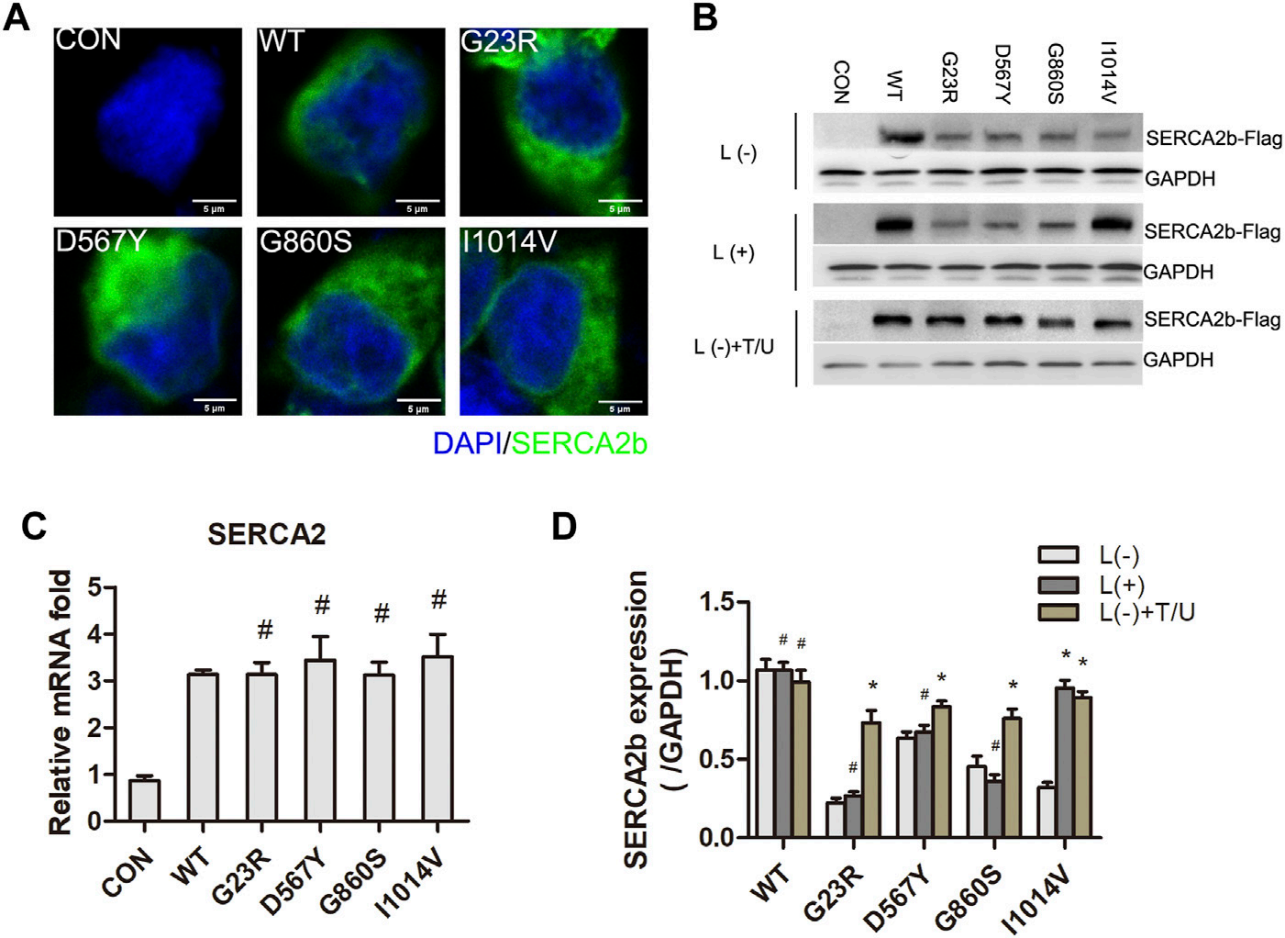
LOF of SERCA2 is caused by insoluble and low-expression of mutants protein. A- B: Intracellular distribution and expression of SERCA2 in mutant cells. Anti-Flag antibody was used to detect the expression of WT and mutant proteins by immunostaining **(A)** and Western Blotting **(B)**. L: lactacystin, T/U: Cells were treated with an additional 1% Triton-X100 and ultrasonic fragmentation. Scale bar, 5 µm. **(C)**: Expression level of SERCA2 mRNA by qPCR. ^#^ *p* > 0.05 compared with WT group, respectively. **(D)**: Quantification of SERCA2 protein level with or without lactacystin and T/U treatment. *p < 0.05 compared with L (-) treatment cells in each groups, ^#^ *p* > 0.05 compared with L (-) treatment cells in each groups, respectively.

We examined the expression and intracellular distribution of exogenously expressed SERCA2 protein by Flag tags. Immunofluorescence results showed that SERCA2 was widely distributed in the cytoplasm (Figure 3A). Compared with the WT group, the exogenous SERCA2 protein expression was significantly reduced in the mutant groups (Figures 3B,D), and this reduction was not significantly associated with the regulation of mRNA transcript levels because qPCR results confirmed that the transcript levels were unaffected (Figure 3C). Further reasons for the decrease in SERCA2 expression could be proteasome-mediated degradation or the formation of aggregates. The aggregates are insoluble in conventional protein lysis buffers, resulting in reduced soluble proteins separated by SDS-PAGE gel. To confirm these two possibilities, we first examined SERCA2 expression after treating cells with the proteasome inhibitor lactacystin (L). As shown in Figures 3B,D, lactacystin treatment did not increase SERCA2 protein expression levels in the WT/G23R/D567Y/G860S groups, indicating that these mutant proteins were not degraded by the proteasome, while partial degradation of the I1014V group was apparent. We then improved the protein lysis treatment process (e.g., adding 1% TritonX-100 to enhance lysis, repeated ultrasonic fragmentation, and denaturation at 98°C extended to 10 min). These results showed that the soluble fraction of the protein was significantly increased compared to that treated by conventional lysis methods (Figures 3B,D), suggesting that some missense mutations may be expressed at levels similar to WT proteins but form insoluble aggregates, which may be related to misfolding of the mutants.

### SERCA2b Mutants Increase Mitochondrial-ER Contacts Leading to Mitochondrial Ca^2+^ Overload and Dysfunction Under Hypo-Glutamate Stimulation

**FIGURE 4:**
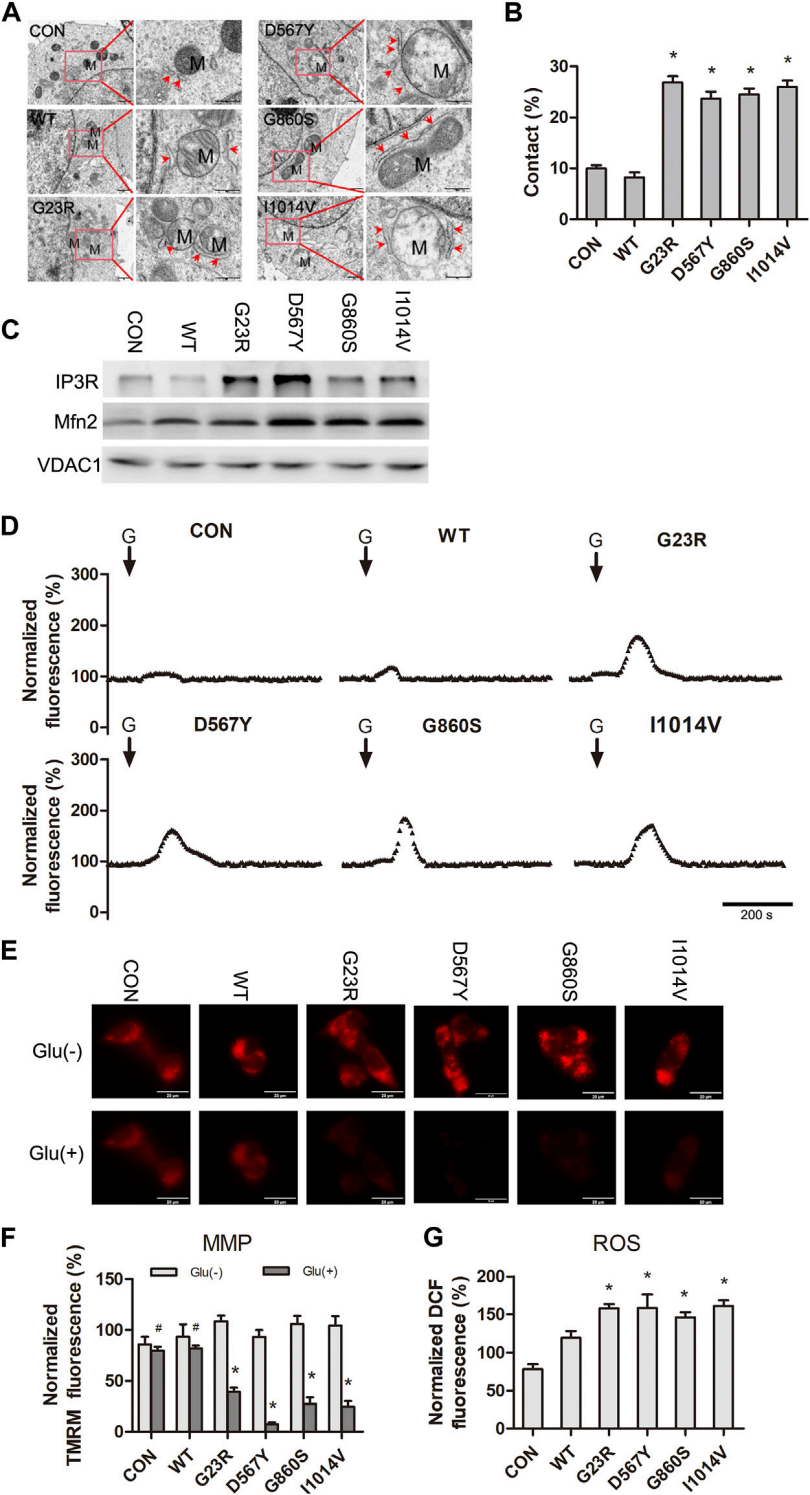
SERCA2 mutants increase mitochondrial-ER contacts leading to mitochondrial Ca^2+^ overload and dysfunction under hypo-glutamate stimulation. **(A)**: Representative electron micrographs of mitochondria-ER contact *SERCA2* mutants. M: mitochondria; red arrows indicate ER. The scale bar is 1 µm (left line), 500 nm (right line). **(B)** Quantification of the mitochondrial surface percentage in close apposition to the ER. *p < 0.05 compared with WT group. **(C)**: Increasing of the calcium channel protein (IP3R and Mfn2) from specifically isolated MAM fractions in *SERCA2* mutants by Western Blotting. **(D)**: Representative normalized fluorescence of mitochondria Ca^2+^ by Rhod-2AM. Scale bar, 200 s. **(G)**: glutamate. **(E,F)**: Decreased mitochondrial membrane potential in *SERCA2* mutants. Representative immunostaining **(E)** and quantification **(F)** of TMRM fluorescence. *p < 0.05 compared with no-glutamate treatment. ^#^ *p* > 0.05 compared with no-glutamate treatment. **(G)**: Increased level of ROS in *SRECA2* mutants by DCFH immunostaining. *p < 0.05 compared with WT group.

Mitochondria are closely associated with the ER through a physical structure, the mitochondria-associated membrane (MAM), which provides energy for the assembly, folding, and modification of ER and the uptake of Ca^2+^ and lipids transferred from the ER. These contact sites are enriched with specific proteins, such as IP3R, Mitofusin 2 (Mfn2), and voltage-dependent anion channel 1 (VDAC1). After confirming that ER Ca^2+^ uptake is impaired in SERCA2b mutants, we wondered whether the mitochondrial function is altered and involved in hypo-glutamate-induced excitotoxicity. Following experiments, we discovered an increased proportion of mitochondrial-ER contacts in the mutants compared to WT group cells by transmission electron microscopy (Figures 4A,B). WB results of isolated MAM fractions showed that the expression of IP3R and Mfn2 was significantly higher in the mutant groups than in the WT group (Figure 4C), suggesting that these proteins may be redistributed from the ER or mitochondrial membrane to the MAM. These results demonstrated that mitochondrial-ER contact sites were increased in SERCA2 mutant cells.

The increased mitochondrial-ER contact and calcium channel proteins of the MAM were the most likely causes of the increased Ca^2+^ flux to mitochondria, resulting in mitochondrial Ca^2+^ overload. We then assessed the kinetics of mitochondrial Ca^2+^ by specifically labeling mitochondrial Ca^2+^ with Rhod-2 AM. Subsequently, mitochondrial Ca^2+^ fluorescence showed a strong response to hypo-glutamate stimulation in the four mutants, followed by a return to the resting levels (Figure 4D). These results suggested that SERCA2b mutants are more prone to mitochondrial Ca^2+^ overload in response to excitatory events. The mutants’ mitochondrial membrane potential (MMP) was significantly decreased after glutamate stimulation (Figures 4E,F), and no significant changes were observed in the WT group. In addition, ROS levels were increased in the mutants compared to the WT group (Figure 4G). These results further confirmed that SERCA2b inactivation causes an increased MAM Ca^2+^ flux associated with mitochondrial dysfunction.

### CDN1163 Inhibits Hypo-Glutamate-Induced Excitotoxicity by Reducing SERCA2 Mutant-Mediated ER Ca^2+^ Depletion and Stress

**FIGURE 5:**
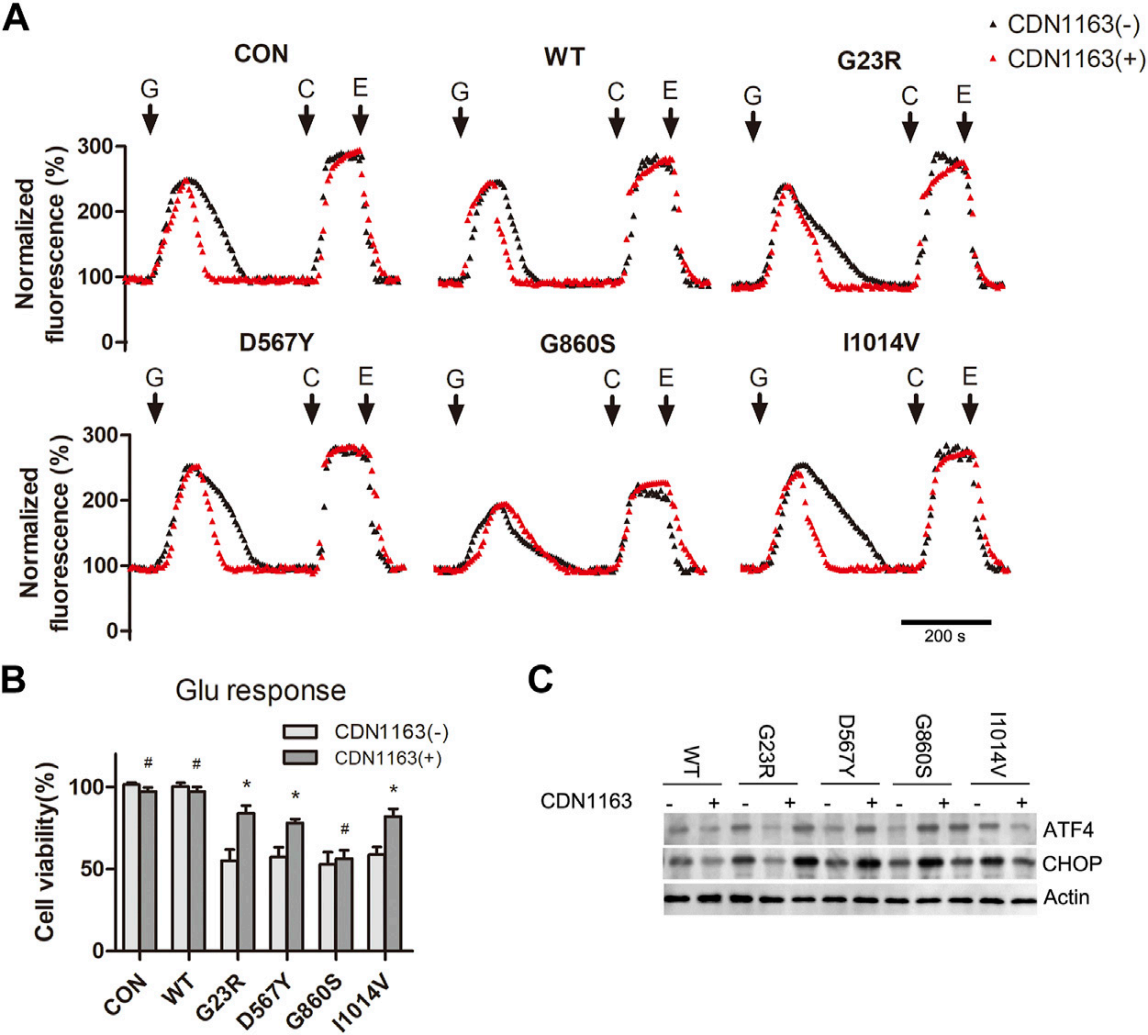
CDN1163 inhibits hypo-glutamate-induced excitotoxicity by reducing SERCA2 mutant-mediated ER Ca^2+^ depletion and stress. **(A)**: Representative normalized fluorescence of cytoplasmic Ca^2+^ by Furo-4 AM. CDN1163 enhanced cytoplasmic Ca^2+^ clearance of SERCA2 mutants. The addition of glutamate (G), caffeine (C), EGTA (E) is indicated with black arrows. Scale bar, 200 s. **(B)**: CDN1163 rescues the cell viability of SERCA2 mutants under hypo-glutamate loading. *p < 0.05 compared with no-CDN1163 treatment, ^#^ *p* > 0.05 compared with no-CDN1163 treatment. **(C)**: Low protein level of ERS marker in SERCA2 mutants with CDN1163 treatment by Western blotting.

Glutamate-induced CICR causes a rapid increase in the levels of [Ca^2+^]_cyto,_ and SERCA2 mutants fails to remove Ca^2+^ effectively, resulting in sustained [Ca^2+^]_cyto_ overload and ER Ca^2+^ depletion. CDN1163 is a small-molecule trans-activator of SERCA that improves intracellular Ca^2+^ homeostasis. We aimed to attenuate glutamate-induced excitotoxicity by increasing the Ca^2+^ capacity of SERCA2. The G23R/D567Y/I140V groups (CDN1163 intervention groups) showed shortened recovery time from the [Ca^2+^]_cyto_ oscillations compared to the group without CDN1163 intervention (Figures 5A,B), suggesting enhanced Ca^2+^ uptake capacity of ER. The efficiency of [Ca^2+^]_cyto_ clearance was unaffected in the G860S group. CCK-8 assay results showed that cell activity in the G23R/D567Y/I140V groups was significantly increased after CDN1163 intervention (Figure 5B), accompanied by a decrease in ERS markers (Figure 5C), while the G860S group did not show significant changes (Figures 5B,C). Taken together, CDN1163 inhibited hypo-glutamate-induced excitotoxicity by reducing mutant-mediated ER Ca^2+^ depletion and stress.

### Inhibition of Pump Leakage Reduces ER Ca^2+^ Depletion and Attenuates Hypo-Glutamate-Induced Excitotoxicity

**FIGURE 6:**
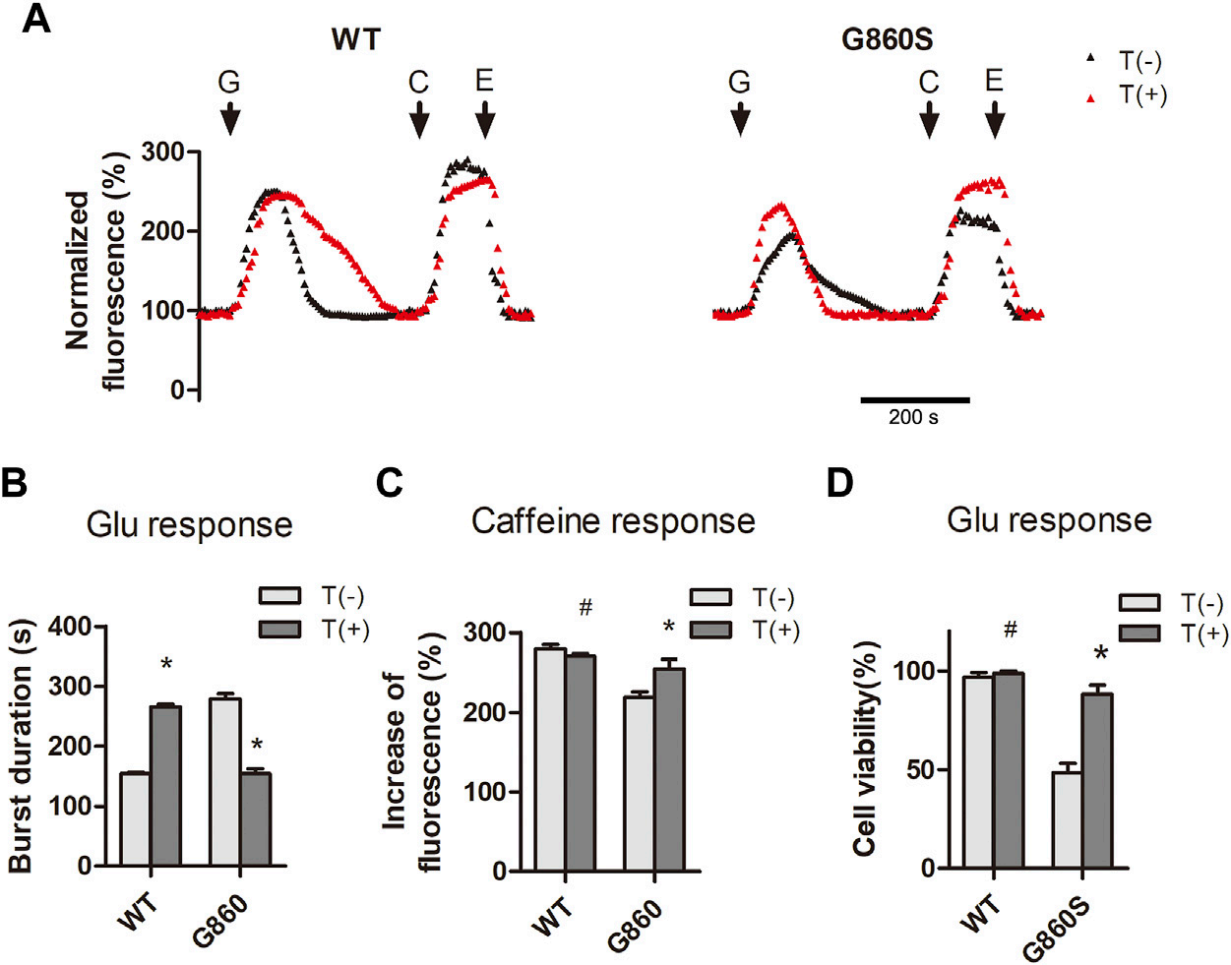
Inhibition of GOF-type pump leakage reduces ER Ca^2+^ depletion and attenuates glutamate-induced excitotoxicity. **(A–C)**: Thapsigargin inhibits pump leakage of G860S mutant. Representative normalized fluorescence of cytoplasmic Ca^2+^ by Furo-4 AM **(A)**. The addition of glutamate (G), caffeine (C), and EGTA (E) is indicated with black arrows. **(B)**: Quantification of the [Ca^2+^]_cyto_ clearance efficiency in response to glutamate. T: Thapsigargin. **(C)**: Quantification of the [Ca^2+^]_ER_ storage in response to caffeine. **(D)**: Thapsigargin rescues the cell viability of the G860S mutant under glutamate loading. (B–D)*p < 0.05 compared with no-thapsigargin treatment, ^#^ *p* > 0.05 compared with no-thapsigargin treatment.

Interestingly, in the above experiments, CDN1163 neither enhanced the [Ca^2+^]_cyto_ clearance efficiency of G860S nor restored the ER Ca^2+^ storage. We speculate that the G860S mutation may lead to the coupling mistake of the SERCA2 gated-channel structure and the acquisition of cis-concentration “leakage capacity.” When the cis-concentration leakage capacity is greater than the inverse “pumping capacity,” the total ER Ca^2+^ flux is toward the cytoplasmic side, leading to the depletion of Ca^2+^ storage, which may be one of the mechanisms of the gain-of-function (GOF) pathogenesis. To confirm this hypothesis, WT and G860S cells were treated with thapsigargin before glutamate stimulation. Thapsigargin is a non-competitive inhibitor of SERCA and does not affect the activity of other ATPases. Calcium signal imaging results showed that thapsigargin reduced the glutamate response time of G860S and partially restored ER Ca^2+^ storage (Figures 6A–C). In contrast, thapsigargin prolonged the [Ca^2+^]_cyto_ clearance of glutamate response in WT-group cells, which is consistent with our previous hypothesis. CCK-8 assay suggested that cell viability in the G860S group significantly increased after thapsigargin intervention (Figure 6D). Taken together, the inhibited leakage of SERCA2 mutants could attenuate ER Ca^2+^ depletion and reduce glutamate-induced excitotoxicity.

## Discussion

Excitotoxicity refers to neuronal damage and death due to chronic or excessive exposure to excitatory amino acids, particularly glutamate, the primary neurotransmitter in mammals. The concept of excitotoxicity was introduced following Olney’s observation of neuropathic damage in rodent and primate brains after systemic injection of glutamate and its analogs (Olney, 1969). Excitotoxicity is widely recognized as a critical player in the occurrence of numerous acute/chronic neurodegenerative pathologies (Ikonomidou and Turski, 1995; Binvignat and Olloquequi, 2020). Although the activation of glutamate receptors leads to the inward flow of different ions, there is a consensus that Ca^2+^ plays a crucial role in excitotoxicity (Choi, 1987), and mitochondrial dysfunction is a critical deleterious effect of excitotoxicity (Mira and Cerpa, 2021). Several clinical trials and animal studies have aimed to address the mechanisms involved in excitotoxicity (Olloquequi et al., 2018; Zaitsev et al., 2020), focusing on the functional effects of glutamate receptors/transporters on cell membranes. These studies brought new questions, such as the memory impairment, hallucinogenic, schizophrenic, and potentially addictive effects of NMDAR antagonists (Allen and Ivester, 2017); the enhancement of the neuronal apoptotic effects (Monti and Contestabile, 2000; Parsons and Raymond, 2014); and the actual relevance of EAAT as a target of pharmacological intervention, which remains to be fully understood and validated (Zaitsev et al., 2020). Given the complex mechanisms of excitotoxicity and the brain’s susceptibility to progressive and long-term damage, there is a need to delve into the underlying mechanisms and facilitate the development of new and effective treatments. The current study investigated the impact of four SERCA2b mutants carried by epileptic patients who suffered from a typical excitotoxic pathogenic disease.

The SERCA2b mutants (G23R, D567Y, G860S, and I1014V) exacerbated Ca^2+^ depletion in the ER under excitatory loading, as evidenced by delayed [Ca^2+^]_cyto_ removal or reduced Ca^2+^ storage. A severe ER Ca^2+^ depletion is an upstream event in the pathophysiology of many neurological diseases. Conversely, impaired ER Ca^2+^ release may no longer maintain essential cellular functions. However, depletion of [Ca^2+^]_ER_ causes ERS and activates UPR, which depends on the duration and severity of the ERS. The mechanism of ER Ca^2+^ depletion is inextricably linked to the functional state of the Ca^2+^ channels of the ER membrane (SERCA, RYR, and IP3R). In the GM1 gangliosidosis model, GM1 accumulates in the microstructure domain of the MAM, which increases ER Ca^2+^ depletion and Ca^2+^ flux to the mitochondria by interacting with phosphorylated IP3R, ultimately leading to mitochondrial Ca^2+^ overload-mediated apoptosis (Sano et al., 2009). In glucocerebroside disease, overactivation of RyR indirectly mediates ER Ca^2+^ depletion, and the redox sensor of RyR may be involved (Lloyd-Evans et al., 2003). In neuropathic pain models, the reduction in SERCA2 leads to decreased ER Ca^2+^ storage, and the resulting disruption of CICR and protein synthesis may contribute to the development of neuropathic pain (Gemes et al., 2009). Unlike the pathogenic mechanism of ER Ca^2+^ depletion in this study, some researchers have suggested that the increased ER Ca^2+^ storage by amyloid oligomers results in the remodeling of Ca^2+^ signaling, which would lead to early learning and memory deficits in the onset of AD (Berridge, 2010).

Intracellular Ca^2+^ regulatory mechanisms can buffer excitatory stimuli from low glutamate concentrations and maintain intracellular homeostasis. However, for SERCA2 mutant cells, this excitatory stimulus is a devastating signal, mainly attributed to the LOF effect due to abnormal SERCA2 expression or activity. Many studies have confirmed that different SERCA2 mutations lead to LOF effects in pump defects or reduced protein levels that underlie dominant diseases. Gordon-Smith et al. found a significantly higher rate of disrupting *ATP2A2* mutations in cases with Darier’s disease accompanied with neuropsychiatric disorders (Gordon-Smith et al., 2018). Ahn’s study confirmed proteasome degradation in the SERCA2 protein (K542X, Q790X, and E917X) (Ahn et al., 2003). However, increased proteasome degradation was also observed in missense mutants (S920Y and I1014V). In addition, a heterozygous splicing mutation in *ATP2A2* also resulted in a shift code and an early termination codon (Nakamura et al., 2016). In addition, Wang et al. discovered that the SERCA2 mutant underwent protein aggregation and formed insoluble aggregates. We performed enhanced treatments on cell lysates in our experiments and confirmed the decreased solubility of SERCA2 mutants. Notably, interactions between SERCA2b monomers were found to dimerize in Ahn’s study (Ahn et al., 2003), affecting the activity of each other, which explains why the pump function of WT is inhibited when WT and mutants coexist. The complicated regulation mechanism of SERCA2 fundamentally affects the differences in local and global Ca^2+^ release kinetics of the mutants.

Only the ER Ca^2+^ storage of G860S was decreased in this experiment. Moreover, the activation of the SERCA2 by CDN1163 failed to rescue ER Ca^2+^ storage. Moreover, the addition of thapsigargin, which is an irreversible SERCA pump inhibitor, did not prevent calcium removal in the G860S mutant. These results indicate that other pathways are implicated in the observed decrease of [Ca^2+^]_cyto_ and uptake by the ER, such as the calcium leakage in G860S mutant or insufficient concentration of thapsigargin. Ion pumps can be thought of as two gated-controlled ion-selective channels, with the gates facing different sides of the membrane. The ATPase enzyme cycle controls the gates and ensures the unidirectional transport of ions. The ability of both gates to open simultaneously is critical to prevent the passive transport of ions through the trans-membrane region. When a minor defect disrupts the coupling between the enzymatic cycle and gating properties, this uncoupling may lead to passive leakage of ions to form GOF channels (Gadsby, 2009). Indeed, SERCA2 can form leaks for Ca^2+^ under certain conditions. For example, the A617T mutation of mSERCA causes ion leakage under heating conditions, and M494L and R131Q also cause ion leakage even without heating, while heating exacerbates the degree of leakage (Kaneko et al., 2014). The marine toxin palytoxin disrupts the coordination of the two gates in the Na^+^/K^+^ ATPase, leading to ion leakage (Artigas and Gadsby, 2003). Ultimately, ER Ca^2+^ storage depends on the balance between the cis-concentration leakage capacity and inverse pumping capacity. In the present study, thapsigargin may have increased ER Ca^2+^ storage primarily through attenuating calcium leakage from G860S. In addition, the concentration window of thapsigargin in inhibiting SERCA pumping or leakage requires further research.

The link between the ER and mitochondria is considered to be highly dynamic, as local Ca^2+^ ions can regulate the ER-mitochondria linkage in different ways (García-Pérez et al., 2008). The increased Ca^2+^ blocks the motility of both organelles and enhances their interactions (Yi et al., 2004), leading to an increase in the contact sites between the MAM and ER, which may be the main reason that SERCA2b mutants lead to mitochondrial disorders under glutamate excitatory loading. MAM is a crucial determinant of cell function and survival, achieved by regulating intracellular Ca^2+^ signaling. MAM dominates the efficient transferring of Ca^2+^ from the ER to the mitochondria, and mitochondrial Ca^2+^ oscillations play a significant role in energy production by regulating calcium-dependent enzymic reactions that generate ATP (Hajnóczky et al., 1995; Rizzuto et al., 2004). Thus, they control the fundamental processes of energy production and determine the cell’s fate by triggering or preventing apoptosis (Green and Kroemer, 2004; Walter and Hajnóczky, 2005). However, MAM is enriched with different functional enzymes involved in lipid metabolism, glucose metabolism, redox reactions, and chaperone molecule folding (Lebiedzinska et al., 2009; Hayashi et al., 2009). Moreover, MAM contains critical Ca^2+^ handling proteins of two organelles (Giorgi et al., 2009; Decuypere et al., 2011). SERCA2 mutants exacerbate mitochondrial dysfunction under excitatory loading by affecting the function of the MAM (Figure 4). Oxidative stress dysregulation is another important factor in mitochondrial dysfunction under excitatory loading. Increased [Ca^2+^]_cyto_ or impaired clearance leads to nitric oxide synthase activation for nitric oxide synthesis, which inhibits mitochondrial electron transport, results in increased production of ROS, and causes a wide range of toxic oxidative responses (Moncada and Erusalimsky, 2002). In excitotoxicity, protein dysfunction, lipid peroxidation, DNA breakage, inhibition of mitochondrial respiratory chain enzymes, and disruption of energy synthesis may occur at any time once the intracellular accumulation of reactive nitrogen species and ROS exceeds the maximum load of the neuronal antioxidant system (Martínez-Ruiz et al., 2011). Therefore, new technologies targeting subcellular delivery of drugs have great potential to rescue mitochondrial dysfunction (Sun et al., 2019).

Different SERCA2b mutants lead to ER Ca^2+^ depletion and mitochondrial dysfunction in response to excitatory events. It is noteworthy that part of the reason is the decrease in pumping capacity (LOF type) and the other factor is the increase in leakage (GOF type), which determines the difference in the scheme of restoring ER Ca^2+^ homeostasis. It is also true that CDN1163 enhances the activity of LOF-type pumps (G23R, D567, and I1014V) to inhibit glutamate-induced excitotoxicity, but not the GOF-type mutant (G860S) (Figure 5). Genetic and phenotypic heterogeneity leads to differences in regimens to treat gene-related disorders. For example, in *SCN2A*, patients with GOF mutations (increased sodium channel activity) respond well to sodium channel blockers (SCBs), whereas patients with LOF (decreased channel availability and membrane excitability) mutations experience exacerbated seizures due to SCB use (Wolff et al., 2017). Notably, the severity of the *ATP2A2* mutant phenotypes parallels the preserved SERCA2 pump activity (Ahn et al., 2003), suggesting that enhancing SERCA2 pump activity and stabilizing ER Ca^2+^ storage are vital to the treatment of excitotoxicity-related disorders. For example, in a neuropathic pain model, enhanced SERCA2 expression or activity in dorsal root ganglia neurons alleviated mechanical and thermal abnormalities in pain, and the reduction of ERS was accompanied by morphological and functional recovery (Li et al., 2022). CDN1163 increased ER Ca^2+^ content, rescued ERS-induced neuronal death *in vitro*, and showed significant efficacy in a 6-hydroxydopamine-induced Parkinson’s disease rat model (Dahl, 2017). The dystrophic phenotype of dystrophin-null transgenic mice was ameliorated by CDN1163, which effectively prevented exercise-induced muscle damage and restored mitochondrial function (Nogami et al., 2021).

**FIGURE 7:**
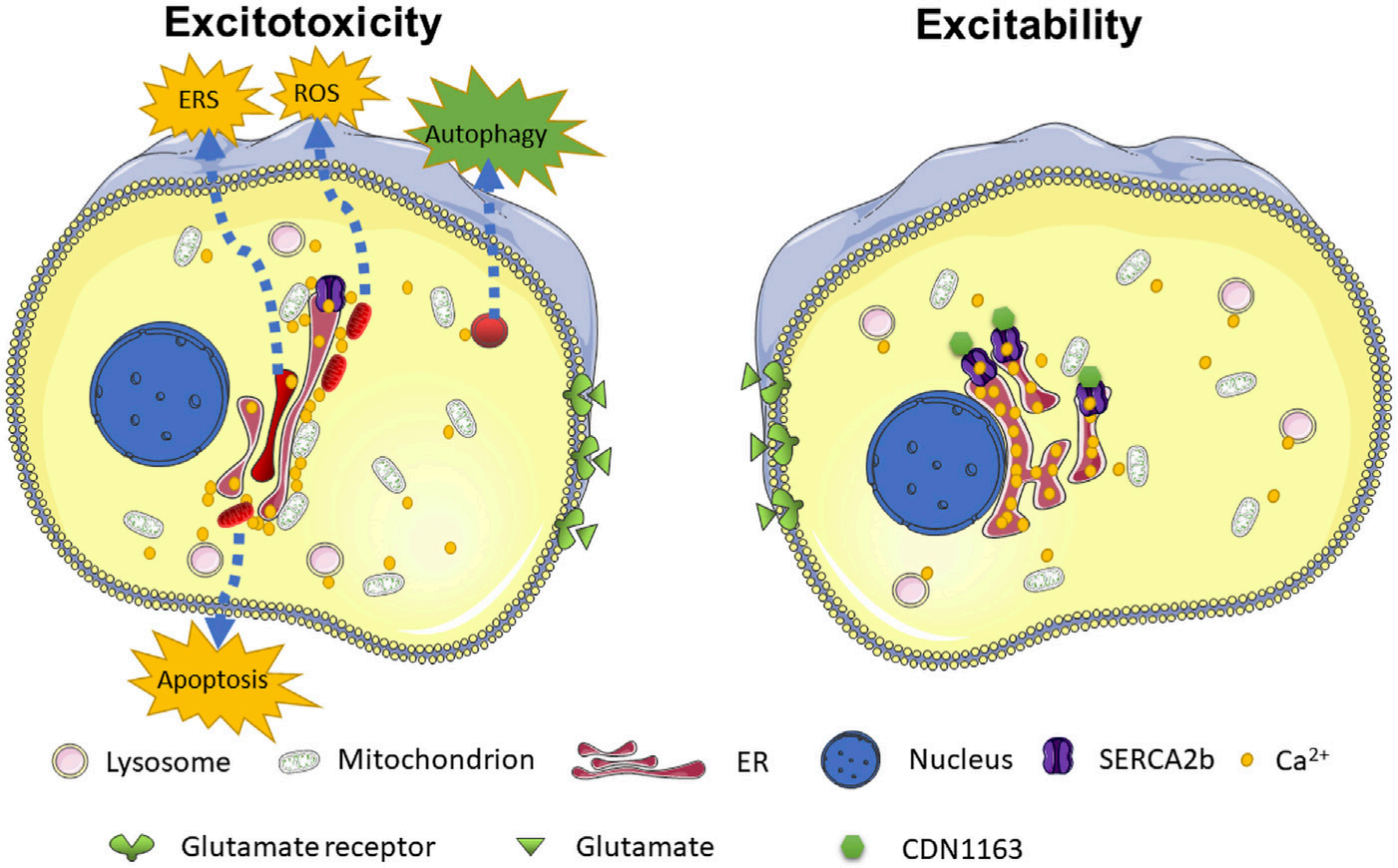
Schematic model depicting the ER Ca^2+^ depletion in response to glutamate-induced excitotoxicity. SERCA2b mutants increase susceptibility to glutamate-induced excitotoxicity by exacerbating ER Ca^2+^ depletion. ER Ca^2+^ depletion leads to increased levels of the [Ca^2+^]_cyto_ and ER-mitochondria contact, which in turn leads to abnormal activation of many Ca^2+^-dependent pathways, such as ERS, oxidative stress, apoptosis, autophagy, and ultimately promotes cell death. CDN1163 enhances SERCA2b activity and inhibits ER Ca^2+^ depletion, maintaining intracellular Ca^2+^ homeostasis. The affected dysfunctional mitochondria, lysosomes, and ER are highlighted in red.

Organelles are in a delicate and dynamic homeostasis conducted by Ca^2+^. As depicted in Figure 7, ER Ca^2+^ depletion caused by SERCA2b mutations disrupts this balance, leading to the activation of a series of cell-death signaling pathways. Targeted treatments against organelles may be a potential approach to synergistically enhance the neuroprotective function of glutamate receptor inhibitors or glutamate transporters (Wang K. et al., 2022; Wei et al., 2022). For example, recovery of the mitochondrial function shows potential therapeutic strategies for traumatic brain injury (Cheng et al., 2012) and neurodegenerative diseases (Cunnane et al., 2020). The suppression of ERS also mitigates epileptic behaviors of the seizure model (Yokoi et al., 2015; Zhu et al., 2017) and AMPA-induced cognitive impairment in rats (Bhardwaj et al., 2021). In recent years, another essential Ca^2+^ storage-related organelle, the lysosome, has gradually been emphasized with excitotoxicity (Vucicevic et al., 2020; Davis et al., 2021). Furthermore, enhanced cytoprotective autophagy can mitigate glutamate-induced excitotoxicity. We may have long overlooked that other cations, such as zinc ions, may play an essential role in AMPK-mediated excitotoxicity (Granzotto et al., 2020; Kim et al., 2020). New ion-tracing techniques, such as ultrahigh-resolution optical microscopy, are promising approaches that can be used to unravel these mysteries (Chen Q. et al., 2020; Fang et al., 2021; Wang L. et al., 2022).

## Conclusion

The current study demonstrates that SERCA2 mutants exacerbate the excitotoxicity of hypo-glutamate stimulation on HT22. SERCA2 mutants accelerate ER Ca^2+^ depletion by either LOF (reduced pumping capacity) or GOF (acquired ion leakage), leading to ERS. The occurrence of ER Ca^2+^ depletion increases MAM formation, contributing to mitochondrial Ca^2+^ overload and dysfunction. Rescuing the SERCA2 pumping capacity or inhibition of Ca^2+^ leak attenuates Ca^2+^ depletion and inhibits excitotoxicity in response to hypo-glutamate stimulation. The stabilization of SRECA2b function is a critical therapeutic target against glutamate-induced excitotoxicity. These data will expand understanding of organelle regulatory networks and facilitate the discovery and creation of drugs against excitatory/inhibitory imbalance in the CNS.

## Data Availability

The original contributions presented in the study are included in the article/Supplementary Materials, further inquiries can be directed to the corresponding authors.
