# Supplementary material for: Restoration of Sarco/Endoplasmic Reticulum Ca2+-ATPase Activity Functions as a Pivotal Therapeutic Target of Anti-Glutamate-Induced Excitotoxicity to Attenuate Endoplasmic Reticulum Ca2+ Depletion
Source: Front Pharmacol. 2022 Apr 20;13:877175. doi: 10.3389/fphar.2022.877175 (PMC9065279; doi:10.3389/fphar.2022.877175)
Supplement: Supplementary file 1 [file DataSheet1.docx]

Supplementary Materials

**Table 1. List of primers used for site-directed mutagenesis PCR**

| Mutants | Forwad Sequence (5' -> 3') | Reverse Sequence (5' -> 3') |
| --- | --- | --- |
| SERCA2b-G23R | GTCAACGAGAGTACGAGGCTGAGCCTGGAACAG | CTGTTCCAGGCTCAGCCTCGTACTCTCGTTGAC |
| SERCA2b-D567Y | CCTGGCCACTCATTACAACCCACTGAGAAG | CTTCTCAGTGGGTTGTAATGAGTGGCCAGG |
| SERCA2b-G860S | ATTGCTGCTGACAGTGGTCCAAGAGTGT | ACACTCTTGGACCACTGTCAGCAGCAAT |
| SERCA2b-I1014V | CACCGATGGGGTTTCCTGGCCG | CGGCCAGGAAACCCCATCGGTG |

**Table 2. List of primary antibodies used in this work**

| Primary antibody | Supplier | (Cat. Number) |
| --- | --- | --- |
| Anti-Flag | CST | #14793 |
| Anti-CHOP | CST | #5554 |
| Anti-ATF4 | CST | #11815 |
| Anti-Mfn2 | Abcam | ab124773 |
| Anti-IP3R | CST | #8568 |
| Anti-VDAC1 | Abcam | ab15895 |
| Anti-SERCA2 | Abcam | ab3625 |
| Anti-GAPDH | Abcam | ab9485 |
| Anti-beta Actin | Abcam | ab8227 |
| Secondary Antibodies for mice | Jackson | #115-035-146 |
| Secondary Antibodies for rabbit | Jackson | #115-035-144 |

Table 3. List of primers used for qPCR

| Gene | Forwad Sequence (5' -> 3') | Reverse Sequence (5' -> 3') |
| --- | --- | --- |
| SERCA2 | TCTAGTTGCTCTGGCTACCAGGCG | CACAGGGCTAAGCACTCCCACTACTT |
| ATF4 | TCGATGCTCTGTTTCGAATG | GGCAACCTGGTCGACTTTTA |
| CHOP | GCATGAAGGAGAAGGAGCAG | CTTCCGGAGAGACAGACAGG |
| Actin | GATCTGGCACCACACCTTCT | GGGGTGTTGAAGGTCTCAAA |
| GAPDH | AGGTCGGTGTGAACGGATTTG | TGTAGACCATGTAGTTGAGGT |
